# Combined treatment with bone marrow mesenchymal stem cells and methylprednisolone in paraquat-induced acute lung injury

**DOI:** 10.1186/1471-227X-13-S1-S5

**Published:** 2013-07-04

**Authors:** Huang Yang, Yin Wen, Yu Hou-you, Wang Yu-tong, Liu Chuan-ming, Xiong Jian, Hao Lu

**Affiliations:** 1 Department of Emergency Xijing Hospital, the Fourth Military Medical University, No.127 Changle Xilu Road Xi'an 710032, People’s Republic of China

## Abstract

**Background**

To evaluate the efficacy of combined treatment with bone marrow mesenchymal stem cell (BMSC) transplantation and methylprednisolone (MP) to treat paraquat (PQ)-induced acute lung injury.

**Materials and methods**

A total of 102 female rats were randomly divided into five groups: PQ, BMSC, MP, BMSC + MP and normal control. After 14 days of PQ poisoning, the survival of rats, wet/dry weight ratio of lung tissue, serum levels of tumor necrosis factor (TNF)-α, interleukin (IL)-1β, IL-6, IL-10, malondialdehyde (MDA) and superoxidase dismutase (SOD), and the expression of nuclear factor (NF)-кB p65 in lung tissue were determined.

**Results**

Rats in BMSC and BMSC + MP groups survived. BMSC transplantation significantly decreased the wet/dry weight ratio of lung tissue, down-regulated NF-кB p65 expression in lung tissue, lowered serum levels of TNF-α, IL-1β, IL-6 and MDA, and increased serum levels of IL-10 and SOD. These changes were particularly significant on days 7–14 after PQ poisoning. The above changes were more significant in the MP group on days 1–3 after PQ poisoning, compared with those of the BMSC group. However, the BMSC + MP group showed more significant changes on days 1–14 after PQ poisoning than those of both BMSC and MP groups.

**Conclusions**

MP inhibits the inflammatory response, reduces the products of lipid peroxidation and promotes survival of transplanted BMSC, thus improving the intermediate and longer term efficacy of BMSC transplantation for treatment of PQ-induced lung injury.

## Introduction

Paraquat (PQ) is an organic heterocyclic herbicide and defoliant agent used worldwide. However, PQ is highly toxic to humans and animals and no effective antidote is available, which causes a high mortality rate[1,2]. Large scale clinical observations and experimental research have shown that the lung is the main target organ for PQ poisoning. This is because pulmonary cells actively take up and accumulate PQ [3-5]. Although patient survival is enhanced by comprehensive therapy, mortality is not obviously improved. Therefore, we are in an embarrassing situation: clinical treatment is still difficult even though the mechanism of PQ poisoning is being studied in more and more depth. Therefore, identifying new therapeutic strategies may help to open this bottleneck. Bone marrow mesenchymal stem cells (BMSC) are an ideal cell source for tissue repair and are increasingly used to treat lung injury. It has been demonstrated that BMSCs migrate into injured lung and, either directly or indirectly, repair damaged lung tissue [6-8]. In this study, BMSC transplantation was combined with methylprednisolone (MP) to treat PQ-induced acute lung injury, and the efficacy of this treatment was evaluated.

## Materials and methods

### Materials

Ethics approval for the study was obtained from the Fourth Military Medical University. Sprague-Dawley(SD) rats (2 male, 120g±20g and 102 female, 200g±20g) were provided by the Experiment Animal Center of the Fourth Military Medical University(Xi’an, China) . 200g/L PQ solution dichloride was obtained from Tianjin Shipule Agriculture Pesticides Technology & Development Inc(Tianjin, China). MP was obtained from Pfizer Inc (New York, USA) . Fluorescein isothiocyanate(FITC)-conjugated anti-mouse CD34, CD45, CD44 and CD166 antibodies were obtained from Biolegend(San Francisco, USA). The nucleoprotein extraction kit was obtained from Promega (Madison, USA), the NF-кBp65 polyclonal antibody from Santa Cruz (California, USA). The rat Interleukin-1β(IL-1β) and Tumor necrosis factor-α(TNF-α) and Interleukin-6(IL-6) and Interleukin-10(IL-10) ELISA kits from Sigma (St.Louis,USA). Malonaldehyde (MDA) and superoxide dismutase (SOD) kits were obtained from the Nanjing jiancheng Bioengineering Institute(Nanjing, China).

### Isolation, culture and identification of BMSC

Male rats were sacrificed by cervical dislocation and the femur and tibia were harvested. The bone marrow cells were gently resuspended into single cells. BMSCs were isolated by Percoll gradient centrifugation, and resuspended in Dulbecco’s modified Eagle’s medium (DMEM)-F12 supplemented with 10% fetal bovine serum Cells were seeded in 25 cm^2^ culture flasks at 1 × 10^5^ cells/cm^2^ and maintained at 37°C with 5% CO_2_ in a humidified incubator. Culture medium was exchanged after 48 h and then every 2–3 days. Cells were cultured to 80–90% confluence and then digested with 0.25% trypsin containing 0.02% EDTA for 1 min. Then, cells were centrifuged, resuspended and passaged at a 1:3 ratio. Third passage cells were tested using flow cytometry for cell surface expression of CD34, CD45, CD44 and CD166 [9].

### Grouping

Female SD rats were randomly divided into five groups: 1) PQ group (n = 24): PQ solution was intraperitoneally injected at a dose of 20 mg/kg. 2). BMSC group (n = 24): BMSCs (0.5 mL, 1 × 10^7^ cells) were injected via the tail vein at 6 h after PQ injection. 3). MP group (n = 24): MP (20 mg/kg) was administered via the tail vein once a day for 2 consecutive days at 2 h after PQ injection. 4). BMSC + MP group (n = 24): BMSCs and MP were administered as described above. 5) Normal control group (n = 6): rats were intraperitoneally injected with 0.9% saline.

### Macro observation

Animals were observed for general activity, feeding and survival for 14 days after PQ poisoning.

### Wet/dry weight ratio of lung tissue

At 1, 3, 7 and 14 days post-injection, the right lung was isolated and the trachea ligated. After blotted off the blood and other contaminants, the wet weight was measured. Then the lung was dried in a 70°C oven for 72 h and the dry weight was measured. The lung wet/dry weight ratio was calculated.

### Determination of cytokine levels

Plasma was collected on days 1,3,7 and 14 post-injection and the concentrations of TNF-α, IL-1β, IL-6, IL-10, MDA and SOD measured by respective ELISAs kits.

### Nucleoprotein extraction and Measurement of NF-кBp65 expression

On days 1, 3, 7 and 14 post-injection, the left-sided lung tissues were isolated, and rinsed with cold phosphate buffer solution (PBS). Nucleoprotein lytic/extraction buffer solution A (2 ml) was added and the tissues incubated on ice for 30 min. The tissue was then homogenized, mixed with 500μl of 10% Nonidet P-40 and centrifuged. The precipitate was rinsed twice with cold PBS and the suspension was discarded. Nucleoprotein extraction/lytic solution B (2 mL) was added to the precipitate on ice for 30 min and the solution centrifuged again to harvest the nucleoproteins. Nucleoproteins (10μg) were measured by western blotting. The images were scanned using a gray-scale scanner and the averaged gray values were used to determine the NF-кBp65 expression level.

### Statistical analysis

All data were statistically analyzed by SPSS 11.5 software and expressed as mean±standard deviation. The 2-group comparison were performed with t-test. P<0.05 was considered statistically significant.

## Results

### *In vitro* culture and identification of BMSC

Adherent cells initially showed round morphology and became spindle- or polygonal-shaped after 3–5 days. Primary cells proliferated as colonies and reached 80% confluence after 10–12 days. Cells showed accelerated proliferation after passaging and were mostly spindle-shaped, with a small portion of polygonal-shaped cells. Flow cytometry showed that 69.4% of 3^rd^ generation cells expressed CD44, 80.7% expressed CD166, and the expression of CD34 and CD45 was absent, indicating that adherent cells were BMSC.

### General conditions and survival of rats

In the PQ group, rats showed reduced activities and feeding, and an increased respiratory rate. Mean survival time was 9.6 ± 1.8 days, ranging from 8–12 days. The MP group did not develop these symptoms until day 3, and the mean survival time was 12.1 ± 1.3 days, ranging from 10–13 days. Therefore, data from the both groups could not be obtained on day 14 after PQ poisoning. All rats in BMSC group and BMSC + MP group survived up to day 14 after PQ poisoning, and the symptoms in BMSC + MP group were significantly improved compared with those of the BMSC group.

### Wet/dry weight ratio of lung tissue

The wet/dry weight ratio of lung tissue in the PQ group was significantly increased compared with that of the control group on day 7 after PQ poisoning (P < 0.01). The wet/dry weight ratio of lung tissue in the BMSC group was similar to that of the PQ group on days 1–3 (P > 0.05), but was significantly decreased compared with that of the PQ group on day 7 (P < 0.01). On days 1–7 after PQ poisoning, the wet/dry weight ratio of lung tissue in the MP group was significantly lower than that of the BMSC group (P < 0.05). The wet/dry weight ratio of lung tissue in the BMSC + MP group was similar to that of the MP group on day 1 (P > 0.05), but was significantly lower than that of the MP group on days 3–7 (P < 0.01). On days 1–14 after PQ poisoning, the wet/dry weight ratio of lung tissue in the BMSC + MP group was significantly lower than that of the BMSC group (P < 0.01) (Table 1).

**Table 1:** Wet/dry weight ratio of rat lung tissue (n = 6)

Groups	1d	3d	7d	14d
PQ	4.51 ± 0.18^a^	5.52 ± 0.21^a^	7.62 ± 0.26^a^	
BMSC	4.51 ± 0.34*^b^*	5.49 ± 0.42*^b^*	6.15 ± 0.42^c^	5.65 ±0.57
MP	4.42 ± 0.29*^d^*	5.32 ± 0.37*^d^*	5.94 ± 0.28*^d^*	
BMSC + MP	4.40 ± 0.51*^ef^*	5.18 ± 0.19*^eg^*	4.79 ± 0.31*^eg^*	4.71 ±0.25 *^e^*
Normal control	4.32 ± 0.31			

Note: ^a ^*P* < 0.01, *vs.* normal control group; *^b^P* > 0.05, ^c ^*P* < 0.01, *vs.* PQ group; *^d^ P* < 0.05, *^e^ P* < 0.01, *vs.* BMSC group; *^f^P* > 0.05, ^g ^*P* < 0.01, *vs.* MP group.

### Plasma levels of TNF-α, IL-1β, IL-6 and IL-10

Plasma levels of TNF-α, IL-1β, IL-6 and IL-10 in the PQ group were significantly elevated on day 1 after PQ poisoning (P < 0.01), compared with that of the control group. On days 1–14 after PQ poisoning, plasma levels of TNF-α, IL-1β and IL-6 in the BMSC group were significantly lowered compared with those of the PQ group, and particularly on days 3 and 7 (P < 0.01). On day 1 after PQ poisoning, IL-10 level in the BMSC group was similar to that of the PQ group (P > 0.05), but was significantly higher than that of the PQ group on day 3 (P < 0.01). The significantly lowered TNF-α, IL-1β and IL-6 levels, and a significantly elevated IL-10 level were found in the MP group compared with those of the BMSC group on day 1 after PQ poisoning (P < 0.01). The significantly lowered TNF-α, IL-1β and IL-6 levels, and a significantly elevated IL-10 level were found in the BMSC + MP group compared with those of the MP group and the BMSC group on day 7 (P < 0.01). (Table 2).

**Table 2:** Plasma levels of cytokines ( ng/L, n = 6)

Group	Post-poisoning day	TNF-α	IL-1β	IL-6	IL-10
PQ	1	83.13 ± 10.29 ^a^	30.25 ± 4.68 ^a^	35.47 ± 2.31 ^a^	18.62 ± 5.46 ^a^
	3	121.51 ± 10.85 ^a^	48.90 ± 4.62 ^a^	98.15 ± 9.44 ^a^	47.86 ± 7.51 ^a^
	7	245.63 ± 12.11 ^a^	102.63 ± 8.71 ^a^	145.61 ± 15.38 ^a^	66.24 ± 5.83 ^a^
	14				
BMSC	1	79.66 ± 8.31 ^c^	21.05 ± 6.47 ^c^	31.37 ± 5.42 *^d^*	18.17 ± 4.63 *^b^*
	3	105.35 ± 8.32 ^c^	43.11 ± 5.15 ^c^	82.40 ± 7.16 ^c^	59.36 ± 7.27 ^c^
	7	144.84 ± 10.73 ^c^	68.78 ± 4.32 ^c^	121.27 ± 9.54 ^c^	83.42 ± 8.03 ^c^
	14	65.20 ± 6.64	21.89 ± 3.56 ^a^	84.53 ± 9.07 ^a^	108.05 ± 10.16 ^a^
MP	1	51.17 ± 7.33 *^e^*	15.62 ± 4.81 *^e^*	19.28 ± 8.66 *^e^*	29.75 ± 8.09 *^e^*
	3	89.15 ±8.65 *^e^*	31.72 ±6.18 *^e^*	70.50 ±8.13 *^e^*	65.39 ±5.24
	7	125.63± 10.44*^e^*	50.64 ±5.74 *^e^*	118.69 ±10.37	85.17 ±7.36
	14				
BMSC + MP	1	50.43 ± 9.61 *^ef^*	13.53 ± 4.17 *^ef^*	17.11 ± 5.36 *^ef^*	27.91 ± 6.39 *^ef^*
	3	67.47 ±9.25 *^e^*	29.56 ±4.52 *^ef^*	71.73 ±8.25 *^ef^*	70.69 ±10.42 *^e^*
	7	71.84 ±8.11 *^eg^*	35.18 ±4.83 *^eg^*	103.68 ±7.70 *^eg^*	92.38 ±6.17 *^eg^*
	14	49.11 ±7.62 *^e^*	14.56 ±2.95 *^e^*	49.27 ±10.34 *^e^*	117.41± 8.82 *^e^*
Normal control		32.08 ± 3.13	10.16 ± 2.56	8.70 ± 0.93	10.04 ± 1.96

Note: ^a ^*P* < 0.01, *vs.* normal control group; *^b^P* > 0.05, ^c ^*P* < 0.01, *vs.* PQ group; *^d^ P* < 0.05, *^e^ P* < 0.01, *vs.* BMSC group; *^f^P* > 0.05, ^g ^*P* < 0.01, *vs.* MP group.

### Plasma levels of MDA and SOD

Plasma levels of MDA in the PQ group were significantly elevated, and SOD levels were significantly decreased compared with those of the control group (P < 0.01). On days 3–7 after PQ poisoning, the significantly lowered MDA levels, and a significantly elevated SOD level were found in the BMSC group, compared with those of the PQ group (P < 0.01).Plasma levels of MDA and SOD in the MP group were significantly lowered and elevated, respectively, on day 1 compared with those of the BMSC group (P < 0.01). MDA and SOD levels in the BMSC + MP group were significantly lowered and elevated, respectively, compared with those of BMSC group and MP group on days on days 3–7 (P < 0.01) (Table 3).

**Table 3:** Plasma levels of MDA and SOD (mean ± SD, n = 6)

Group	MDA (nmol/ml)				SOD (U/ml)			
	
	1d	3d	7d	14d	1d	3d	7d	14d
PQ	4.03 ±0.61 ^a^	4.65 ±0.78 ^a^	5.07 ±0.64 ^a^		287.66 ±11.53 ^a^	246.32 ±16.31 ^a^	212.58 ±19.22 ^a^	
BMSC	3.97 ±0.70 *^b^*	4.32 ±0.48 ^c^	4.16 ±0.81 ^c^	3.63±0.56	285.17 ±13.69 *^b^*	261.24 ±20.74 ^c^	269.74 ±15.64 ^c^	284.42± 17.56
MP	3.82 ±0.58 *^d^*	4.49 ±0.46	4.06 ±0.62		294.52 ±10.14 *^d^*	265.43 ±15.26	280.15 ±14.58	
BMSC + MP	3.78 ±0.74	4.20 ±0.29 *^de^*	3.79 ±0.51 *^de^*	3.31±0.68	292.38 ±9.06	278.66 ±11.84 *^de^*	302.37 ±10.11 *^de^*	304.15± 10.63
Normal control	3.06 ± 0.24				308.29 ± 18.83			

Note: ^a ^*P* < 0.01, *vs.* normal control group; *^b^P* > 0.05, ^c ^*P* < 0.01, *vs.* PQ group; *^d^ P* < 0.01, *vs.* BMSC group; ^e ^*P* < 0.01, *vs.* MP group.

### Expression of NF-кB p65 in lung tissue

NF-кB p65 expression in lung tissue from the PQ group was significantly elevated and peaked on day 3 after PQ poisoning, compared with that of the control group (P < 0.01). NF-кB p65 expression from the BMSC group was lowered on day 1 (P < 0.01), compared with that from the PQ group. NF-кB p65 expression in the MP group was significantly lowered on days 1–3, compared with that in the BMSC group (P < 0.01), but was similar to that from the BMSC group on day 7 (P > 0.05). The BMSC + MP group showed similar expressions of NF-кB p65, compared with those of the MP group on days 1–3 (P > 0.05), but were significantly lower than those of the BMSC group (P < 0.01). In addition, NF-кB p65 expressions in the BMSC + MP group on days 7-14 after PQ poisoning were lower than those in the BMSC group (P < 0.05) (Table 4).

**Table 4:** Gray values for NF-кB p65 expression in lung tissue (mean ± SD, n = 5)

Group	1d	3d	7d	14d
PQ	139.54 ± 12.46^a^	188.78 ± 17.73^a^	140.49 ± 9.21^a^	
BMSC	125.32 ± 10.63*^b^*	146.56 ± 12.55*^b^*	101.84 ± 7.45*^b^*	95.77 ± 8.12
MP	116.47 ± 8.91 ^c^	127.82 ± 10.39^c^	103.37 ± 11.44*^e^*	
BMSC + MP	115.15 ± 10.48^cf^	125.16 ± 9.83^cf^	96.41 ± 12.32^d^	89.75 ± 7.60^d^
Normal control	82.36 ± 6.53			

Note: ^a ^*P* < 0.01, *vs.* normal control group; *^b^P* < 0.01, *vs.* PQ group; ^c ^*P* < 0.01, *^d^ P* < 0.05, *^e^ P* > 0.05, *vs.* BMSC group; *^f^P* > 0.05, MP group.

## Discussion

Alveolar cells actively uptake and accumulate PQ causing severe lung injury. The consensus of the poisoning mechanism is changes in redox potential. PQ activates NF-кB and promotes the expression and release of early inflammatory mediators. In addition, PQ produces large amounts of oxygen free radicals, induces the production of lipid peroxides and causes direct damage to major cell components [10-12]. Dinis-Oliveira et al [13] found that NF-кB is constantly elevated within 96 h after intraperitoneal injection of PQ, which is consistent with the findings of this study. We found that NF-кB is an oxidation-stress sensitive transcription factor in PQ-induced lung injury. Significant and sustained activation of NF-кB during days 1-7 after PQ poisoning causes the release of inflammatory cytokines(TNF-α, IL-1β, IL-6, IL-10), initiating a cascade effect and resulting in uncontrollable lung inflammation. In addition, large amounts of MDA were produced, which reduced free radical scavenger SOD and caused cell damage.

Stem cell therapy has gained a great amount of attention in basic research and clinical application for treatment of lung injuries. Rojas et al. [14] found that bleomycin induces severe lung injury after ablation of mouse bone marrow, and exogenous BMSC transplantation significantly reduces lung inflammation. We found that BMSC transplantation significantly reduced NF-кB p65 expression in lung tissue after PQ poisoning, thus reducing the serum levels of TNF-α, IL-1β and IL-6 resulting in elevated IL-10 levels. In addition, transplantation of BMSCs also reduced MDA levels and SOD consumption, thereby lowering the wet/dry weight ratio of lungs after PQ poisoning. It has been suggested that BMSC reduce edema and exudation in lung injury, inhibit the release of inflammatory mediators and lipid peroxidation, attenuate inflammation and reduce cell damage. We also found that BMSC transplantation showed relatively weak efficacy in reducing NF-кB p65 on day 1 after PQ poisoning, which was consistent with the serum levels of TNF-α, IL-1β, IL-6 and IL-10, suggesting that BMSCs did not efficiently inhibit the early inflammatory response. We speculate that the grafted BMSCs required time to be fully functional after transplantation and therefore were not effective during days 1–3 after PQ poising. However, BMSC transplantation maintained constant protection during days 7–14 after PQ poisoning.

A number of cytokines and inflammatory mediators attract transplanted BMSC, but are also toxic and may compromise the survival of BMSC. It has been shown that TNF-α neutralization decreases the incidence of lung injury in mice after bone marrow transplantation [15,16]. High-dose MP is widely used to treat PQ poisoning because of its potent anti-inflammatory effects and ability to improve early lung injury. However, the use of corticosteroids does not reduce mortality after PQ poisoning [17]. In this study, we showed that during days 1–3 after PQ poisoning, treatment with MP alone was superior to that of BMSC transplantation to reduce NF-кB p65 expression in lung tissue, lower serum levels of IL-1β, TNF-, and IL-6, increase IL-10 levels, inhibit MDA production and SOD consumption and lower the wet/dry weight ratio of lungs. However, the efficacy of MP to treat lung injury gradually decreased on days 1–7 after PQ poisoning, which may explain why high-dose MP improves the symptoms of PQ poisoning, but does not reduce mortality. The efficacy of BMSC transplantation combined with MP for the treatment of PQ-induced lung injury was investigated in this study. We found that combination with MP improves the relatively poor efficacy of BMSC transplantation for the treatment of lung injury during days 1–3 after PQ poisoning. MP inhibited the inflammatory response, reduced the products of lipid peroxidation and promoted the survival of BMSC, thus improving the intermediate and longer term efficacy of BMSC transplantation to treat PQ-induced lung injury.

## Conclusions

In summary, the combination of BMSC transplantation and MP shows an improved protective effect in PQ-induced acute lung injury. However, the mechanism of PQ poisoning are complicated and remain unclear. Further investigation is needed to understand the effects of BMSCs in acute lung injury.

## Declarations

This article has been published as part of *BMC Emergency Medicine* Volume 13 Supplement 1, 2013: Proceedings of the 2012 Emergency Medicine Annual Congress. The full contents of the supplement are available online at http://www.biomedcentral.com/bmcemergmed/supplements/13/S1.

The publication costs for this article was funded by Xijing Hospital, the Fourth Military Medical University.
